# Body dissatisfaction among French and German sports science students – a cross-country comparison

**DOI:** 10.3389/ijph.2026.1610065

**Published:** 2026-10-21

**Authors:** Elke Grimminger-Seidensticker, Gaëlle Le Bot, David Savage, Elodie Chaplais

**Affiliations:** 1 Department of Exercise and Health, Paderborn University, Paderborn, Germany; 2 Inter-University Laboratory for Education and Communication Sciences UR 2310, University of Lorraine, Nancy, France; 3 School of Education and Lifelong Learning, Norwich Research Park, University of East Anglia, Norwich, United Kingdom; 4 Development, Adaptation, Handicap Laboratory UR 3450, University of Lorraine, Nancy, France

**Keywords:** body image, curriculum studies, intercultural comparison, mental health, physical activity

## Abstract

**Objectives:**

This cross-country study examines body (dis)satisfaction in relation to motives for using fitness-related social media among sports science students in Germany and France.

**Methods:**

A total of 523 German (mean age 21.22) and 611 French (mean age 19.85) sports science students completed a questionnaire to assess body dissatisfaction, drive for thinness, drive for size, and motives for engaging with fitness-related social media.

**Results:**

Two-way factorial ANOVAs revealed that French students exhibited significantly greater body dissatisfaction and drive for thinness compared to their German counterparts. French female students demonstrated a stronger drive for size; male French students demonstrated a stronger drive for thinness. Hierarchical regression analysis showed that body image issues were predicted by gender, country, and social media motives, such as seeking dieting ideas.

**Conclusion:**

Qualitative studies are necessary to investigate unconventional body image concerns among French students. Given that future practitioners act as role models and may be responsible for developing health education programs, it is crucial to ensure that they can educate others in a healthy manner.

## Introduction

Physical education (PE) teachers and sports professionals are expected to have physically fit, slim bodies, representing “healthy bodies” [1]. Their bodies function not only as working machines but also as role models for fitness-oriented lifestyles, serving as a prerequisite for professional credibility with no room for aged or ill bodies [2]. Discourse analyses reveal that these messages about sporty, healthy bodies are embodied in pedagogical practices, presented as either “naturally given” physical capital [2] or the result of self-discipline, suggesting that obesity can be avoided [3]. University socialization and lower body self-esteem reinforce these anti-fat attitudes [4]. Finally, PE and health education teacher students, along with health educators and nutritionists, show higher rates of exercise disorders and body image problems compared to students in other disciplines [5–8]. Fitness instructors also report body appearance pressure, negatively affecting their mental health [9], similar to dance students showing high levels of depression, anxiety symptoms, and disordered eating [10]. However, body image issues affect all university students, not only sports science students, as all university students experience pressure regarding body appearance [11].

From a theoretical point of view, body image is a multidimensional construct with three components [12]: 1) Perceptual (mental) representations of body size/shape; 2) Cognitive-affective representations (thoughts/feelings about the body), which involve self-ideal comparisons potentially resulting in body dissatisfaction [13]; 3) The behavioral component, reflecting behavior toward or with the body. For example, individuals exercise or diet to shape their body to achieve the ideal body they aspire to [13]. These body ideals are gender-stereotyped: men should achieve muscular, lean bodies [14] with muscular arms, backs, and chests, broad shoulders, defined abdominals, and narrow waists [15]. Women’s beauty ideal combines slenderness with moderate musculature and a flat belly, well-defined legs, big bottoms, and big chests [16]. These ideals are popular on social media, especially in #fitspiration content [17, 18].

Body image development is influenced by historical/developmental factors and proximal factors [12]. Thus, cultural differences in body image exist, largely driven by societal expectations around mental wellbeing and appearance [19]. As body image is shaped by societal norms and expectations, it can be considered a socially constructed phenomenon regulated by social institutions [20]. Consequently, university sports science programs function as distinct sociocultural environments centered on physical activity, which can foster body image concerns or worsen existing tendencies [21], for example, through someone simply commenting on one’s body. These body images may also be transmitted and legitimized through social media. Thus, body image is historically and societally shaped, resulting in individual evaluation processes and investment behaviors toward and with the body. However, no cross-country studies exist on body image issues among sports science students from two different European countries. Thus, this article compares German and French sports science students to explore the impact of sociocultural context and fitness-related social media consumption on body image perception. This quantitative questionnaire study will explore the following research questions:

1. Are there differences in the level of body image issues (e.g., body dissatisfaction, drive for thinness, and drive for size) between German and French sports science students?
2. Which factors, including country, age, gender, Body Mass Index (BMI), physical activity, and social media use, explain the variation in body image issues?

This study aims to evaluate body image concerns among sports science students to develop curriculum measures that promote wellbeing by focusing on body diversity rather than unrealistic body ideals [9].

## Methods

### Participants

In Germany, 670 individuals accessed the online questionnaire, and a total of 523 German sports science students could be included in the study (275 men, 238 women, and 4 diverse; mean age: 21.22; response rate: 78.06%). In France, 776 people accessed the online questionnaire, and a total of 611 French sports science students could be included in the study (386 men, 221 women, and 3 nonbinary; mean age: 19.85; response rate: 78.74%. 6 persons with missing sex/gender, and for the French sample: 1 person with missing sex/gender). In Germany, the sample was collected at five different universities: three in western Germany, including the first author’s university, one in south-western Germany, and one in eastern Germany. In France, the sample was collected at the authors’ university in eastern France. Participants surveyed at the respective universities included undergraduate sports science students in their first through third years, graduate students in their first or second years, and those in training to be physical education teachers. Although the students completed the questionnaire during lessons, it was stressed that their participation was voluntary and that non-participation would not have negative consequences. The study was reviewed and approved by an ethics committee, with the approval number 37/2024.

### Instruments

The questionnaire consisted of several different parts focusing on different elements of the study. In the first part, sociodemographic data (gender, age) were obtained. Self-reported weight and height were used to calculate the Body Mass Index (BMI). To measure the level of physical activity, the participants reported how many hours they were physically active per week outside of their university sports courses and whether they were engaged in competitive sports. Social media engagement, specifically about fitness-related content, was measured by self-reported time spent on social media in general per day (in minutes/hours) along with time spent on fitness-related content. The participants’ reasons for using fitness-related social media content were measured using items such as “I use social media to inspire me to improve my body shape, tone or size” [22]. These items were translated from English to German and French and back to English. Different aspects of body image issues were assessed using the “Body dissatisfaction” (BD) and “Drive for thinness” (DFT) scales in the German and French versions of the Eating Disorder Inventory-2 (EDI-2) [23, 24]. “Drive for size” (DFS) was measured using the German and French versions of the Muscle Dysmorphic Disorder Inventory (MDDI) [25, 26]. The three scales are six-point Likert scales ranging from “never” (1) to “always” having this issue (6).

### Data analysis

To investigate the main and interaction effects of gender and country on the three body image scales “Body dissatisfaction” (BD), “Drive for thinness” (DFT), and “Drive for size” (DFS), a two-way factorial ANOVA was conducted. *Bonferroni* was selected for the confidence interval adjustment to hold the error rate for multiple comparisons to *α* = 0.05, and the contrast *repeated* was chosen. Post-hoc analyses were conducted to identify significant differences within the different groups (gender/country). Prior to the main analysis, the statistical assumptions of the model were evaluated. Normality of the residuals was assessed using the Shapiro-Wilk test and visual inspection of normal Q-Q plots. Although the Shapiro-Wilk test indicated a statistically significant deviation from normality (*W*_BD_ (1120) = 0.54), *p* < 0.001; *W*_DFT_ (1058) = .12, *p* < 0.001; *W*_DFS_ (1057) = 0.08, *p* < 0.001), the evaluation of the Q-Q plots revealed no substantial departures from linearity. Given the large sample size and the robustness of the ANOVA framework under the central limit theorem [27], the assumption of normality was considered to be met. Homogeneity of variances was evaluated using Levene's test based on the deviations from the group means. The test indicated significant heterogeneity of variances (*F*_BD_ (3, 1) = 45.71, *p* < 0.001; *F*_DFT_ (3, 1) = 44.23, *p* < 0.001; *F*_DFS_ (3, 1) = 61.50, *p* < 0.001). To control for inflated Type I error rates and preserve statistical power without resorting to data transformation, heteroscedasticity-consistent standard errors were implemented. Specifically, the HC3 estimator was utilized to compute robust standard errors and adjusted p-values for the main and interaction effects, as is recommended for linear models under heteroskedasticity [28].

To identify significant predictors of body image issues, hierarchical multiple regression analyses were conducted. The following predictors were introduced as blocks with the “enter” method: In the first block, country was used as the control variable, followed by gender (female/male) in the second block, BMI in the third block, hours of physical activity per week and engagement in competitive sports (yes/no) in the fourth block, and amount of social media usage in general per week in minutes along with amount of fitness-related social media use per week in minutes in the fifth block. Finally, the reasons for consuming fitness-related social media content were used in the sixth block (in total, 7 items; coded as yes/no^1^).

Missing data were handled using analysis-specific complete-case analysis. Each analysis included only participants with complete data for the variables involved; therefore, sample sizes varied across analyses. No missing values were imputed. All analyses were calculated with IBM® SPSS® Statistics Version 21.

## Results

A descriptive analysis showed that 93.8% of German and 97.1% of French sports science students exercise regularly outside of their university’s timetabled sports seminars. Only 0.4% of German and 0.5% of French students do not access social media, while 12.3% of German and 8.9% of French students do not access fitness-related social media content. Table 1 provides an overview of the mean scores on the three body image scales, separated by country and gender.

**TABLE 1:** Mean score and standard deviation of body image issue scales for German and French Sports Science Students in (Image Corporelle des Étudiants en Sciences du Sport: une étude comparatiste entre la France, l'Allemagne et l'Angleterre, Germany and France, 2024–2025).

Scale	Germany		France	
​	Male	Female	Male	Female
Body dissatisfaction (mean, SD)	2.52 (0.45)	2.85 (0.49)	2.89 (0.51)	3.01 (0.48)
Drive for thinness (mean, SD)	1.78 (0.75)	2.52 (1.22)	2.49 (0.98)	2.78 (1.05)
Drive for size (mean, SD)	2.82 (1.05)	1.80 (0.75)	2.94 (1.17)	2.74 (1.07)

All three scales are 6-point Likert scales, with 1 = never, 2 = rarely, 3 = sometimes, 4 = often, 5 = normally and 6 = always.

To determine how many students frequently experience body image issues, we calculated the percentage of the sample who answered higher than “sometimes”. The results are displayed in Table 2.

**TABLE 2:** Relative porportions of students in the sample who experienced body image issues more than “sometimes” (Image Corporelle des Étudiants en Sciences du Sport: une étude comparatiste entre la France, l'Allemagne et l'Angleterre, Germany and France, 2024–2025).

Scale	Germany		France	
​	Male	Female	Male	Female
Body dissatisfaction	13.5%	31.4%	31.8%^1,2^***	37.2%
Drive for thinness	6.8%	28.6.%	22.9%^1,3^***	32.2%
Drive for size	34.7%	7.5%	40.4%	29.1%^1,4^***

^1^differences compared to the same gender group in the other country; ^2^ *χ* ^*2*^ (1) = 26.94, *p* < 0.001, *C*_*corr*_ = .44; ^3^ *χ* ^*2*^ (1) = 28.16, *p* < 0.001, *C*_*corr*_ = .45; ^4^ *χ* ^*2*^ (1) = 34.87, *p* < 0.001, *C*_*corr*_ = .52; ***p < 0.001.

Table 3 provides an overview of the descriptive data included in the hierarchical regression models.

**TABLE 3:** Overview of the descriptive data included in the hierarchical regression models (Image Corporelle des Étudiants en Sciences du Sport: une étude comparatiste entre la France, l'Allemagne et l'Angleterre, Germany and France, 2024–2025).

Variable	German students		French students	
​	Male	Female	Male	Female
Mean body Mass index	23.33 (SD = 2.37)	21.72 (SD = 2.27)	22.43 (SD = 2.62)^1,2^***	21.68 (SD = 2.47)
Mean hours of physical activity per week	9.61 (SD = 4.77)	8.31 (SD = 4.00)	12.06 (SD = 6.63)^1,3^***	9.77 (SD = 5.73)^1,4^**
Engagement in competitive sports (yes)	53.2%	52.6%	67.6%^1,5^***	57.4%
Amount of social media usage in general, in minutes per day	195.87 (SD = 89.92)	184.59 (SD = 74.85)	198.80 (SD = 99.30)	205.36 (SD = 111.46)^1,6^*
Amount of fitness-related social media usage in minutes per day	70.34 (SD = 61.84)	52.72 (SD = 51.12)	60.20 (SD = 58.62)^1,7^*	59.04 (SD = 54.18)
Reasons for consuming fitness-related social media content…	​	​	​	​
… to inspire me to exercise to improve my health or wellbeing (yes)	62.3%	64.3%	31.8%^1,8^***	34.6%^1,9^***
… to eat healthy food (yes)	53.0%	71.2%	24.1%^1,10^***	32.7%^1,11^***
… to learn more about health and wellbeing (yes)	47.7%	42.6%	52.0%	56.7%^1,12^**
… to improve my body shape, tone, or size (yes)	28.2%	29.4%	29.4%	33.6%
… to exercise to gain muscle/become stronger (yes)	61.7%	47.5%	34.7%^1,13^***	33.2%^1,14^**
… to diet to lose weight (yes)	8.0%	19.1%	9.0%	17.1%
… to improve my appearance (yes)	23.9%	22.5%	31.3%	39.2.%^1,15^***

^1^differences are calculated within the same gender but different country groups; ^2^ *t* (652) = 4.91, *p* < 0.001, Cohen’s *d* = .23; ^3^ *t* (625) = −5.02., *p* < 0.001, Cohen’s *d* = .41; ^4^ *t* (415) = −3.00, *p* = 0.003, Cohen’s *d* = .29; ^5^ *χ* ^*2*^ (1) = 13.32, *p* < 0.001, *C*_*corr*_ = .32; ^6^ *t* (450) = −2.34, *p* = 0.020, Cohen’s *d* = .22; ^7^ *t* (595) = 2.00, *p* = 0.046, Cohen’s *d* = .17; ^8^ *χ* ^*2*^ (1) = 51.98, *p* < 0.001, *C*_*corr*_ = .52; ^9^ *χ* ^*2*^ (1) = 37.37, *p* < 0.001, *C*_*corr*_ = .54;^10^ *χ* ^*2*^ (1) = 50.47, *p* <0 .001, *C*_*corr*_ = .48; ^11^ *χ* ^*2*^ (1) = 62.55, *p* < 0.001, *C*_*corr*_ = .68; ^12^ *χ* ^*2*^ (1) = 8.29, *p* = 0.004, *C*_*corr*_ = .27; ^13^ *χ* ^*2*^ (1) = 40.10, *p* < 0.001, *C*_*corr*_ = .47; ^14^ *χ* ^*2*^ (1) = 8.96, *p* = 0.003, *C*_*corr*_ = .26; ^15^ *χ* ^*2*^ (1) = 13.48, *p* < 0.001, *C*_*corr*_ = .30; ***p < 0.001; **p < .01; *p < 0.05.

The two-way factorial ANOVAs revealed a significant gender*country interaction effect on all body image scales (BD: (*F* (1, 1062) = 11.73, *p* < 0.001; *partial η* ^*2*^ = 0.011; DFT: (*F* (1, 1057) = 13.17, *p* < 0.001; *partial η* ^*2*^ = 0.012); DFS: (*F* (1, 1056) = 37.45, *p* < 0.001; *partial η* ^*2*^ = 0.034)). Post-hoc analyses revealed that French female students were significantly more dissatisfied (mean = 3.01) with their body than their German counterparts (mean = 2.84; *t* (442) = −3.55, *p* < 0.01, *d* = 0.35). Male French students were also more dissatisfied (mean = 2.78) in comparison to male German students (mean = 2.52; *t* (617) = −9.38, *p* < 0.01, *d* = 0.77). French female students also had a significantly higher drive for thinness (mean = 2.78) than German female students (mean = 2.52; *t* (439) = −2.35, *p* = 0.019, *d* = 0.23). French male students (mean = 2.49) also had a higher drive for thinness in comparison to their German counterparts (mean = 1.77; *t* (615) = −9.71, *p* < 0.01, *d* = 0.82). Regarding drive for size, French female students had a significantly higher desire to become more muscular (mean = 2,73) than German female students (mean = 1,80; *t* (438) = −10.62, *p* < 0.01, *d* = 1.00), while there was no difference between French and German male students.

The hierarchical regression analysis revealed three significant models, one for each body image scale. Thus, body dissatisfaction is significantly predicted by country (France), gender (female), BMI, and the use of fitness-related social media content for inspiration on dieting to lose weight, improving body shape, tone, or size, and eating healthy food. (*R* ^2^ = 0.22; *F* (1, 924) = 4.50; *p* = 0.034) (see Table 4).

**TABLE 4:** Hierarchical regression model of body dissatisfaction (Image Corporelle des Étudiants en Sciences du Sport: une étude comparatiste entre la France, l'Allemagne et l'Angleterre, Germany and France, 2024–2025).

​	​	Body dissatisfaction
*Predictor*	*N*	*β (p)* [95% CI]
​	932	​
Country	​	0.31 (0.00)*** [0.27, 0.40]
Gender	​	0.21 (0.00)*** [0.16, 0.28]
Body Mass index	​	0.19 (0.00)*** [0.03, 0.05]
Amount of social media use in general	​	0.03 (0.25) [0.00, 0.00]
Use of fitness-related social media content… … to inspire me to diet and lose weight … to inspire me to improve my body shape, tone, or size … to inspire me to eat healthy food	​	0.21 (0.00)*** [0.23, 0.43] 0.07 (0.03)* [0.01, 0.15] 0.07 (0.03)* [0.01, 0.15]

***p < 0.001; **p < 0.01; *p < 0.05.

The drive for thinness is significantly predicted by female gender, French nationality, a higher BMI, and the consumption of fitness-related social media content. Specifically, individuals were found to be more likely to pursue thinness if they use this content for inspiration to lose weight, improve their appearance, eat healthily, or build muscle (*R* ^2^ = 0.29; *F* (1, 920) = 4.41; *p* = 0.036) (see Table 5).

**TABLE 5:** Hierarchical regression model of drive for thinness (Image Corporelle des Étudiants en Sciences du Sport: une étude comparatiste entre la France, l'Allemagne et l'Angleterre, Germany and France, 2024–2025).

​	​	Drive for thinness
*Predictor*	*N*	*β (p)* [95% CI]
​	928	​
Country	​	0.23 (0.00)*** [0.37, 0.64]
Gender	​	0.17 (0.00)*** [0.25, 0.51]
Body Mass index	​	0.08 (0.01)** [0.01, 0.06]
Use of fitness-related social media content… … to inspire me to diet and lose weight … to inspire me to improve my appearance … to inspire me to eat healthy food	​	0.36 (0.00)*** [0.95, 1.36] 0.12 (0.00)*** [0.14, 0.44] 0.07 (0.04)* [0.03, 0.31]
… to inspire me to exercise to gain muscle	​	−0.07 (0.04)* [-0.28, −0.01]

***p < 0.001; **p < 0.01; *p < 0.05.

Several distinct factors significantly predict an individual’s drive for body size. These predictors include living in France, being male, and not participating in competitive sports. Additionally, higher levels of both general social media use and consumption of fitness-specific content increase this drive, as does exercising with the specific motivation to gain muscle (*R* ^2^ = 0.20; *F* (1, 918) = 59.08; *p* < 0.001) (see Table 6).

**TABLE 6:** Hierarchical regression model of drive for size (Image Corporelle des Étudiants en Sciences du Sport: une étude comparatiste entre la France, l'Allemagne et l'Angleterre, Germany and France, 2024–2025).

​	​	Drive for size
*Predictor*	*N*	*β (p)* [95% CI]
​	928	​
Country	​	0.21 (0.00)*** [0.34, 0.64]
Gender	​	−0.23 (0.00)*** [-0.68, −0.40]
Age	​	−0.05 (0.12). [0.0.05, 0.01]
Engagement in competitive sports	​	−0.06 (0.04).* [-0.29, −0.01]
Amount of social media use in general	​	0.10 (0.001)** [0.00, 0.002]
Amount of fitness-related social media use	​	0.08 (0.01)** [0.01, 0.04]
… to inspire me to exercise to gain muscle	​	0.24 (0.00)*** [0.41, 0.69]

***p < 0.001; **p < 0.01; *p < 0.05.

## Discussion

The descriptive findings reveal that nearly all German and French sports science students exercise regularly outside of timetabled sports seminars at university. The weekly amount of physical activity ranges from 8.31 h (for female German students) to 12.61 h (for male French students), revealing that French students are significantly more physically active than German students. Over half of the students engage regularly in competitions, with the highest proportion being French men (67.6% of them). Both groups show higher activity levels than national data suggest: 58% of German women and 69.3% of German men (aged 18–29) engage in aerobic exercise for approximately 150 min weekly [29], or 50.3% of French women and 69.1% of French men (18–39) engage in moderate to vigorous physical activity (MVPA) for more than 30 min on more than 5 days per week or more than 25 min MVPA on at least 3 days per week [30].

Social media is widely used by the study participants, with the majority of students accessing social and fitness-related content daily. They spend from 3 h 34 min daily (German women) to 3 h 41 min (French women) on social media, with German men spending the highest amount of time on fitness-related content (1 h 10 min daily). Both groups show similar consumption patterns to those of young European adults (16–29), of whom 97% use the internet daily [31].

Body image issues vary notably between French and German students, though overall dissatisfaction appears lower than in the general, non-sporting population. Although precise levels of body dissatisfaction are difficult to report due to multiple predictors (e.g., BMI, gender, age, ethnicity, sexual orientation) [32], general population data show that 45% of French university students [33] and 41% of young German adults experience body dissatisfaction, with 53% of Germans feeling pressured by beauty standards [34]. The rates among the studied group are lower, ranging from 17% to 21.9% for German students and from 26.3% to 33.7% for French students.

Gender-specific data reveal distinct trends in body dissatisfaction and physical appearance goals: More than a third of French (37.2%) and German female respondents (31.1%) frequently experience body dissatisfaction. The majority of these students desire to be thinner (32.2% of French vs. 28.6% of German women). Additionally, nearly a third of French women in the study frequently wish to have more muscle. French men in the study report higher overall body dissatisfaction (31.8%) compared to German men. However, a desire for increased muscularity is common to both groups, affecting 40.4% of French men and 34.7% of German men.

The relative proportions in our study sample may be lower, as research indicates that engagement in physical activity can influence body satisfaction. It is also possible that our sports science students correspond to the societal body ideal, meaning that, compared to other study populations, their level of body dissatisfaction is lower, or they engage in physical activity to maintain or work on their physical appearance [35].

Regarding the cross-country comparison, it is possible to deduce that French female and male students are significantly more dissatisfied with their bodies than their German counterparts. French female and male students also show a significantly higher drive for thinness than German female and male students. These results align with those of a cross-country study in 12 different countries, in which French female university students had a significantly lower BMI than German female university students, among other findings, and also showed a greater discrepancy between their ideal and actual body image [36]. Despite having a lower BMI than German male students, French male students are significantly more likely to want to lose weight and use exercise to do so—an alarming finding that highlights a potential issue with body image.

Regarding drive for size, there is no difference between French and German male students, while French female students wish significantly more often to have more muscle than German female students. French female students also exercise significantly more than their German counterparts, which could be interpreted as a behavioral change aimed at losing weight and gaining muscle simultaneously.

In both Germany and France, general population studies show that body ideals remain highly gender-specific: women are expected to be skinny, while men are expected to be strong and muscular [37, 38]. However, our study revealed a unique exception among French sports science students: men showed a higher drive for thinness and females showed a higher drive for body size. This contradicts broader Western research, which typically associates slimness with women [14], and a lean, muscular build with men [39]. Indeed, a 12-country study confirms that while 53% of French adults view a slim body as ideal for women, only 37% prefer it for men [38]. This variation suggests that the specific sociocultural environment of French sports science programs creates a unique subculture within Western norms [19]. The differences between French female and German female students regarding their drive for size could also be explained by the male-dominated context of sports studies in France: 55% of sports science students in France are men [40], and in our sample, only 36.2% are women. Thus, French female students may also adhere to masculine body norms that are typically associated with men around increased muscularity dissatisfaction [41]. This interpretation is also supported by the assumption that body ideals are constructed by social institutions [20].

However, twenty-first-century media aimed at men presents unrealistic ideals of leanness and muscularity [42], so that male and female body ideals may become increasingly similar, especially in a target group such as that of sports science students, where the ideal of an ‘athletic body’ that is slim and, at the same time, muscular may emerge. As exposure to appearance-related media content is a key mechanism through which cultural ideals become internalized [19], this trend towards an ‘athletic body’ could also be influenced by body image ideals represented in social media, especially in fitness-related, internationally widespread trends such as #fitspiration, which characterize this ‘athletic body’ as being thin and visibly muscular [43].

The majority of the participants in this study indicated that they access fitness-related content, and they may also internalize those ‘athletic bodies’ as their own beauty ideal. This is supported by our hierarchical regression models (Tables 4-6), revealing that the reasons for using fitness-related content on social media are associated with body image issues. Furthermore, female French students were found to spend significantly more time on social media in general than their German counterparts, while male French students spent significantly more time on fitness-related content on social media than German male students.

Body dissatisfaction is most strongly linked to being female, living in France, having a higher BMI, and using social media to find weight-loss diets. The impact of a higher BMI likely reflects the weight stigma and negative views of larger bodies often found on social media [44], and among sports science students [2]. This bias may explain why body dissatisfaction is significantly higher among individuals with obesity compared to those of normal weight [45]. Furthermore, seeking weight-loss diet information on social media is the strongest predictor of a drive for thinness, followed by French nationality and female gender. However, it is possible that sports science students who are already dissatisfied with their bodies actively seek out fitness-related social media content to change their appearance [22, 46].

The strongest predictor of a drive for thinness is using social media to get inspiration about how to diet and lose weight, followed by country (France) and gender (female). The regression model does not reflect the dual standard of being slim and muscular at the same time, as social media is not used for building more muscle. Therefore, the finding that French female students use social media significantly less to gain muscle than German female students is unexpected and needs further exploration. However, as the items in the “Drive for thinness” scale reflect restrictive eating behavior, this scale can also be implemented to detect eating disorders and is even more sensitive than the severity indices for eating disorders in the Diagnostic and Statistical Manual of Mental Disorders, Fifth Edition (DSM-5) [47]. The relationship between body dissatisfaction is well researched, with three main risk pathways suggested [48]; however, a recent longitudinal twin cohort study revealed that body dissatisfaction may increase disordered eating and depressive symptoms [49]. Recent studies also highlighted the highly negative impact of social media on the development of eating disorders, suggesting that misuse of social media, such as searching for weight-loss strategies, can trigger the development of eating disorders within those who are already struggling with this issue [50].

The primary predictor of the “drive for size” is using social media for muscle-building inspiration, followed by male gender and residing in France. Exposure to muscular ideals on platforms such as Instagram heavily increases men’s drive for muscularity and symptoms of muscle dysmorphia [51]. While the link between social media and the drive for thinness is well-established in female students [52], its impact on their drive for size is less researched [53]. However, viewing thin or lean-and-muscular female bodies significantly increases women’s body dissatisfaction [54], as demonstrated by a cross-sectional analysis of French undergraduate students [55]. Nevertheless, longitudinal data from these French undergraduate students reveal that interpersonal pressure from peers and partners, mediated by appearance comparison, ultimately drives these concerns and the desire for thinness [55]. This suggests that French sports science students may face stronger peer pressure than their German peers.

Consequently, both male and female French sports science students simultaneously pursue thinness and size, arguably creating compulsive behavioral patterns [56]. This dangerous dual ideal of being concurrently thin and toned promotes a risky combination of caloric restriction and intensive strength training, which can cause severe health complications [57, 58].

Although the relative proportion of body image issues in our sample of German and French sports science students is lower than in other reference studies, approximately 30% of the students in each subsample appear to have a form of body dissatisfaction. French male and female students are more affected by the dual attractive standard of being thin and muscular, which appears to pose a potential risk of severe health problems. Also, for both subsamples, we can state that a high level of physical activity alone does not protect against body image issues; from a theoretical point of view, the nature of media exposure and institutional culture may shape body image[12]. However, sports science students and their teachers reproduce interactively valued body norms within the academic environment. Therefore, students and teachers are at the same time “products” and “producers” within an institutional culture [20]. Therefore, it would be beneficial for future studies to obtain the perspectives of those who teach and train the future sports scientists to better comprehend the messages delivered within the sports science curriculum and to build a body-positive academic community.

All forms of body image issues are associated with social media behavior, in which students are confronted with the “athletic body” ideal, a unifying yet problematic standard for sports science students in France and in Germany. Sports science students either develop body image issues due to exposure to this body norm, or they are dissatisfied with their bodies and seek information on social media to change their appearance. A study involving adolescents in 42 countries highlighted that intensive and problematic social media use is linked to negative body image in adolescents, with girls at the highest risk of body dissatisfaction and conflicting thin-muscular ideals [59].

With sports science students serving as professional role models, they need culturally sensitive prevention programs addressing body image issues and disordered eating [19, 53]. To counter the challenges posed by modern social media, these programs should build digital health competence and visual physical literacy [60]. Specifically, training should celebrate diverse body types, abilities, ages, illnesses, and healthy eating habits, while actively addressing the risks of muscularity-driven diets and toxic protein overconsumption [61].

### Strengths and limitations

This study included a large participant base and socioculturally validated measurements to ensure validity and reliability. The author team is interculturally mixed, meaning that the viewpoints are enriched by a variety of perspectives. However, limitations include the cross-sectional design of the study and the need for careful interpretation of the regression models, although they are theoretically based on Cash´s cognitive-behavioral heuristic model [12]. Furthermore, the regression models only explain a modest portion of the variance, suggesting that other relevant predictors may be missing and should be included in future studies. Following the Tripartite Influence Model [62], further studies should also consider the family context, the internalization of societal ideals of appearance, and heightened appearance-comparison tendencies. In addition, some of the predictors included in our study, such as BMI, the amount of time spent on physical activity, and (fitness-related) social media use, rely on self-reported data, which may be inaccurate. The results are also not generalizable due to local sampling in France and small sample sizes at each German university, despite attempts made to cover different regions in Germany. Although we calculated hierarchical regression models, the results should be interpreted as preliminary and hypothesis-generating rather than confirmatory. In particular, future qualitative research with sports science students in France and Germany is needed to understand the mechanisms and processes behind their body image issues and to identify other predictors not yet included in the regression models.

## Data Availability

The datasets presented in this article are not readily available due to ethics and data privacy reasons. Requests to access the datasets should be direct to the corresponding author.

## Footnotes

1 Students who identified as nonbinary or had missing sex/gender data were excluded from the regression analyses.
